# Notch3 Knockout Suppresses Mouse Mammary Gland Development and Inhibits the Proliferation of 4T1 Murine Mammary Carcinoma Cells via CCL2/CCR4 Axis

**DOI:** 10.3389/fcell.2020.594372

**Published:** 2020-11-17

**Authors:** Wei Xiong, Junyu Tan, Yuxian Guo, Shuzhao Chen, Liping Fan, Yaochen Li

**Affiliations:** ^1^The Central Laboratory of Cancer Hospital, Shantou University Medical College, Shantou, China; ^2^Guangdong Provincial Key Laboratory of Breast Cancer Diagnosis and Treatment, Shantou, China

**Keywords:** Notch3, knockout mice, mammary glands development, CCL2/CCR4 axis, intraductal injection

## Abstract

Our previous study found that Notch3 knockout mice exhibit defects in mammary gland development. To elucidate the underlying mechanism, tissue samples were subjected to RNA-seq, GO, and KEGG enrichment analyses and qRT-PCR validation. Of enriched pathways, chemokine signaling pathway and cytokine–cytokine receptor interaction were noticed in both Notch3^wt/wt^/Notch3^wt/–^ and Notch3^wt/wt^/Notch3^–/–^ mice, in which the expression of chemokine ligand 2 (CCL2) was sharply reduced in Notch3^wt/–^ and Notch3^–/–^ mammary gland tissues. The Mouse ENCODE transcriptome data reveal that the mammary gland fat pad exhibits a high CCL2, CCR2, and CCR4 expression, indicating that these molecules play important roles during mammary gland development. Specifically, defective mammary glands in Notch3 knockout mice could be partially rescued by CCL2 overexpression lentivirus through intraductal injection. An *in vitro* study showed that CCL2 overexpression promoted the proliferation, migration, and cancerous acinar formation of 4T1 cells, which could rescue the defective migration of 4T1 cells caused by Notch3 knockdown. We also found that Notch3 transcriptionally regulated the expression of CCL2 in a classical pattern. Our findings illustrated that Notch3-regulating CCL2/CCR4 axis should be an important signaling pathway for mammary gland development and should be a candidate target for breast cancer therapy.

## Introduction

Compared with other major organs, the mammary gland is a unique glandular organ because it reaches full development only after birth. Its development can be divided into three main stages throughout the lifetime, namely, embryonic, pubertal, and adult (Macias and Hinck, 2012). During these different stages, the epithelial cells of the mammary gland will undergo many rounds of proliferation, differentiation, or apoptosis in response to the hormonal changes induced by the estrous/menstrual cycle as well as the dramatic changes that occur during pregnancy, lactation, and involution, giving rise to significant remodeling of the branching morphogenesis or glandular tissue architecture (Inman et al., 2015). Accordingly, the mammary gland provides a distinctive model for biologists to study development, organ specificity. In fact, many dysregulated pathways and malignant biological processes observed in breast cancer progression mimic those observed during normal mammary gland development and tissue remodeling. For instance, Wnt-Cadherin signaling that controls the initial epithelium invasion in the fat pad for the elongation of mammary ducts and regulates their continuing plasticity is very similar to that occurring in the infiltration, migration, and clonogenicity adopted by malignant mammary gland epithelial cells (Meniel and Clarke, 2003). In addition, based on the model system of mouse mammary gland, many researchers also constantly explore developmental mechanisms and some basic questions in cancer biology by knocking in/down certain gene(s), which have been extrapolated to humans (Lanigan et al., 2007).

Notch is a well-known transmembrane receptor in an evolutionarily conserved signaling pathway that plays a crucial role in stem cell maintenance, differentiation, proliferation, motility, survival, tissue patterning, and cell fate specification during development (Nichols et al., 2007). In mammals, the Notch family has four members (Notch1–4) that interact with five Notch ligands (DLL1, DLL3, DLL4, JAG1, and JAG2). Usually, Notch mediates local cell–cell communication and coordinates a signaling cascade present in all animal species studied to date.

Upon ligand binding, Notch receptors are activated by sequential cleavages involving members of the ADAM (a disintegrin and metalloprotease domain) protease family (α-secretases) and γ-secretase. These cleavage events result in the translocation of the Notch intracellular domain to the nucleus, where it engages the DNA-binding protein CBF1/RBP-Jκ, resulting in the replacement of a multiprotein corepressor complex with a coactivator complex, thus initiating the transcription of target genes (Callahan and Egan, 2004).

Like most glandular tissues, different types of cells are found in the mammary glands, including epithelial, adipose, fibroblasts, immune, lymphatic, and vascular cells. These different cell types have been demonstrated to be of importance at specific stages of mammary gland development.

Emerging evidence indicates that Notch signaling plays a critical role in mammary stem cell function, luminal fate commitment (Callahan and Egan, 2004; Bouras et al., 2008), and even in mammary development (Dontu et al., 2004). Recently, Lafkas et al. (2013) showed that Notch3 marks luminal progenitor cells and restricts their proliferation and consequent clonal expansion.

Herein we provide solid evidence that Notch3 is required for mouse mammary gland development by directly regulating the downstream target molecule CCL2 expression at the transcriptional level in a classical Notch signaling pattern. At the same time, we also report that Notch3 knockdown can inhibit the proliferation and the migration of 4T1 murine mammary carcinoma cells *via* the CCL2/CCR4 axis.

## Materials and Methods

### Mice and Reagents

Mice were housed in groups in a specific pathogen-free environment, and experiments were given approval by the Shantou University Medical College Animal Ethics Committee. All experiments conformed to the relevant regulatory standards. Female FVB.129S1(B6)-Notch3^tm1Grid/MshaJ^ mice were used in this study. The mice were purchased from Jackson Laboratory (Bar Harbor, ME, United States).

### Mating and Gestation

Animals 8–10 weeks of age and weighing ≥ 20 g were used for mating. A single male mouse was paired with two females. Because female Notch3^–/–^ mice do not like to nurse the babies, the Notch3^–/–^ males were mated with Notch3^wt/–^ females to generate Notch3^–/–^ females. This procedure was repeated to generate Notch3^–/–^ females. After 4 days of pairing, the males were removed. At birth, each mother/offspring set was maintained in clean filtered air, and the number of litters with viable and dead offspring was determined, respectively. After weaning (3 weeks), the pups and the mothers were separated, and the genotypes of the offspring were tested. All the mice used in this study and their numbers are listed in Supplementary Table S3.

### Genotyping

The offspring were genotyped by performing PCR of the genomic DNA derived from tail clippings. The primers for genotyping are listed in Supplementary Table S1. The PCR programs for genotyping are based on the suggestions provided by The Jackson Laboratory.

### Cell Lines

HEK-293T and 4T1 mouse breast cancer cell lines were purchased from the Committee on Type Culture Collection of the Chinese Academy of Sciences (Shanghai, China). These cell lines had been analyzed by short tandem repeat. The cells were routinely grown with Dulbecco’s modified Eagle’s medium (DMEM) supplemented with 10% fetal bovine serum and 1% penicillin/streptomycin. The cells were passaged every 4–5 days, and the medium was changed once between splits.

### Transcriptome Profiling by RNA-Seq

The mice were euthanized by cervical dislocation. Excised mammary gland tissues were snap-frozen in liquid nitrogen and stored at −80°C until use. Total RNA was isolated from tissues or cells with the TRIzol reagent (Invitrogen, CA, United States), according to the manufacturer’s instructions. Equal amounts of total RNA from three mice with the same genotype were either pooled or individually used. Transcriptome sequencing (RNA-Seq) was performed by the BGI company.

### Real-Time qRT-PCR and Western Blotting

Total RNA was isolated from breast cancer cell lines using TRIzol Reagent. First-strand cDNA was synthesized by incubating 1 μg of total RNA with oligo dT Primer and reverse transcriptase (PrimeScript RT reagent kit with gDNA Eraser, Takara, Kusatsu, Japan), according to the manufacturer’s protocol. The primers are listed in Supplementary Table S1.

To detect protein expression levels, mammary glands tumor tissues were homogenized in ice-cold radioimmunoprecipitation assay lysis buffer (Beyotime, Beijing, China). The homogenates were then centrifuged at 12,000 × *g* for 20 min at 4°C. The protein content in the clear supernatant was quantified using a bicinchoninic acid kit (Beyotime, Beijing, China), and samples were then reduced and stored at −80°C until use. The primary antibodies used in this study are listed in Supplementary Table S2. Details on the experimental methods and data processing are described in the Supplementary Materials and Methods. Student’s *t*-test was used to determine statistical significance. All values are presented as mean ± SD.

### Mammary Glands Whole Mount and Carmine Alum Staining

The mice were euthanized by cervical dislocation. The mammary glands were dissected, spread onto a glass slide, fixed in methanol (10%), dehydrated in graded solutions of ethanol, cleared in xylene overnight, hydrated in graded solutions of ethanol, and stained in carmine alum staining solution [0.2% carmine (Sigma) and 0.5% aluminum potassium sulfate (Sangon Biotech)] for about 2 days until the stain has infiltrated the tissues. Finally, these were dehydrated in graded solutions of ethanol, cleared in xylene overnight, and mounted with a coverslip. The slides were scanned, and digital pictures were taken by Perkin Elmer Vectra Slide Analysis System which was powered by inForm^®^ software (BS, MT, United States).

### Morphometric and Thresholding Analysis

In detail, the whole mounted mammary glands were viewed through a Zeiss Stemi 2000-c dissection scope at ×3.2. Images were captured by an AxioCam HRc digital camera (Zeiss) at 2,600 dots per inch (dpi) and processed with Axiovision software (version 4.3; Zeiss) to measure the following parameters: ductal tree; ductal extension, measured as the distance from the nipple to the furthest point of growth; number of branching points; number of secondary and tertiary branches; number of terminal ends; and primary duct length, measured as the distance from the nipple to the first branching point. The method is widely used in mammary development biology.

### Intraductal Injection for Localized Gene Delivery to the Mouse Mammary Glands

The mice were divided into two groups, control and experimental group, and anesthetized using 1% pentobarbital sodium (0.1 ml/20 g). A protocol for the non-invasive intraductal delivery of aqueous reagents to the mouse mammary glands is described and shown from the website https://www.jove.com/video/50692/intraductal-injection-for-localized-drug-delivery-to-mouse-mammary. It is important to enter the nipple slightly past the bevel of the needle (not more than 2 mm) to prevent penetration through the mammary tissue into the serous membranes of the ventral body cavity. The needle should be inserted into the nipple slightly deeper than 3 mm, slowly drawing the syringe back up out of the duct until 2 mm from the tip of the bevel so that the mammary tissues were ensured to be targeted instead of the muscle surrounding the peritoneal cavity of mice. Moreover, visualization of the injection is difficult, and practice for this step is needed. We recommended practicing this step by injecting trypan blue and preparing whole mammary mounts to confirm targeting of the ductal tree as well as using the non-transgenic mice until the injection technique is optimized. Specifically, we postponed the injection time and allowed the nipples and the primary duct to develop as much as possible so that the nipples can be seen clearly under a stereoscope.

### *In vivo* Bioluminescence Imaging of Mammary Glands

Bioluminescence was monitored using an IVIS200 imaging system (PerkinElmer IVIS Lumina). At 12 days after the CCL2 ectopic overexpression, lentivirus was intraductally injected. D-Luciferin [15 mg/ml, dissolved in phosphate-buffered saline (PBS)], the substrate of luciferase, was injected intraperitoneally for a working dose of 10 mg/kg, and the mice were anesthetized (isoflurane, 2.5%).

Bioluminescence (photons/s) was then acquired every 5 min after the injection. The obtained images were analyzed with the Living Image Software.

### Cell Proliferative Assay

Cell proliferation potential was evaluated by cell proliferation assays using CCK-8 assays (Sigma). The 4T1 cells ectopically expressing CCL2 were plated at a concentration of 500 cells/ml into 96-well culture plates. The cells transfected with empty vectors were used as negative controls. The medium that contained 10% FCS was used as blank control. Replications were done with five wells plating the same cells. For the cell proliferation potential assay, 10 μl of CKK-8 solution was added to each well of the plate at 0, 1, 2, 3, and 4 days after inoculation, and then the plates were incubated for 2 h. Absorbance was then measured at 450 nm using a microplate reader (SpectraMax M5, Sunnyvale, CA, United States).

### Spheroid Formation Assay

4T1 cells were transfected with EX-Mm05119-Lv217 and EX-Mm05119-Lv217/CCL2 lentivirus, respectively. The cells were treated with trypsin, respectively, and first resuspended in complete medium (DMEM medium supplemented with 10% FBS and 1% penicillin/streptomycin). The cells were pelleted through centrifugation at 125 × *g* for 5 min and then resuspended in complete medium. Cell concentration was counted by the TC20 automated cell counter (Bio-Rad). Then, 100 μl of cell suspension containing 1 × 10^5^ cells and 200 μl Matrigel matrix (10 mg/ml) (Corning) were mixed, the mixture of which was kept on ice. Pre-chilled tips were used to seed the mixture into a 24-well plate. The plate was incubated at 37°C for 30–45 min. Each well was gently added with 500 μl complete medium. The culture was kept for 8–10 days, and the complete medium was changed every 2 days for further observation of malignant acini development along a time course.

### Chromatin Immunoprecipitation

Chromatin immunoprecipitation (ChIP) was performed as previously described, with minor modifications (Saleh et al., 2008). To determine whether the Notch3 regulate the expression of CCL2, mouse myoblast C2C12 cells were grown on a medium containing 10% FBS, and the medium was replaced to inducing medium which contained 1% horse serum for 2 days. Cross-linked chromatin was sheared, by sonication using a Biosafer250-88 Ultrasonic homogenizer (Biosafer, Nanjing, China), five times for 5 s and an interval of 10 s with a microtip in a 1.5-ml tube. The supernatant from the irrelevant antibody served as a positive control (“input,” 1% of the ChIP material). Chromatin was immunoprecipitated with Notch3 antibody. Immunoglobulin G (IgG) was used as a negative control. Protein A+G agarose was added to the antibody/chromatin complex, and the mixture was incubated overnight at 4°C. The protein A+G agarose–antibody/chromatin complex was resuspended in wash buffer and centrifuged to collect the protein/DNA complex. Protein/DNA crosslinks were reversed to obtain free DNA. The purified and immunoprecipitated DNA was analyzed by semi-quantitative PCR. The ChIP primer sequences are provided in Supplementary Table S1.

### Immunohistochemical Staining

The samples were fixed with neutral formalin, embedded in paraffin, and sectioned at a thickness of 4 μm. The sections were deparaffinized in xylene and rehydrated in a graded alcohol series. Antigen retrieval was performed, and hydrogen peroxide was applied to block endogenous peroxidase activity. Non-specific protein binding was blocked. The sections were incubated overnight at 4°C with primary antibodies (Supplementary Table S1) and were stained in parallel with non-immune IgG as a negative control. The expression levels and patterns of target proteins were then detected using an EliVision Plus kit (Maixin, Fuzhou, China). 3,3′-Diaminobenzidine visualization was then performed, and the slides were counterstained with hematoxylin. The slides were randomized and examined by two independent investigators. Five views were examined per slide, and 100 cells were observed per view at ×400 magnification.

### Colony Formation Assay

The mouse triple negative breast cancer 4T1 cells were inoculated at 1 × 10^5^ cells/ml in a six-well plate and allowed to attach for 24 h. The cells were then respectively transfected with 2.5 μg of EX-Mm05119-Lv217/CCL2 or empty vector EX-Mm05119-Lv217, using Endofectin transfection reagent. At 48 h post-transfection, the transfected cells were reseeded into six-well plates at a density of 500 cells/well and incubated for 14 days. At the end of the experiment, the cells were fixed with 4% paraformaldehyde for 60 min and then washed with PBS and stained with 500 μl of 1% gentian violet, and the number of colonies was counted and analyzed. The protocol was reported previously. The experiment was independently repeated three times. All values are presented as mean ± SD.

### Wound Healing Assay

First, the 4T1 cells grown in 10-cm dishes were transfected with a reagent mixture composed of 15 μg of EX-Mm05119-Lv217/CCL2 or empty vector EX-Mm05119-Lv217 (diluted in 750 μl serum-free medium) and 45 μl Endofectin transfection reagent (diluted in 750 μl serum-free medium). Then, the transfected cells were incubated until 90% confluency. The layer of cells was scratched with a 2-mm-wide pipette tip and washed with PBS three times. Incubation was continued for some time in a medium containing 1% FBS DMEM. An average of five random widths along the injury line was measured for quantitation. All values are presented as mean ± SD.

### Statistical Analysis

Statistical analysis was performed using GraphPad prism 6 software. The results are presented as means ± SD. Statistically significant differences were calculated using Student’s *t*-test. *P*-value < 0.05 was considered significant.

## Results

### Notch3 Is Required for Ductal Morphogenesis of Mouse Mammary Gland Development

**FIGURE 1:**
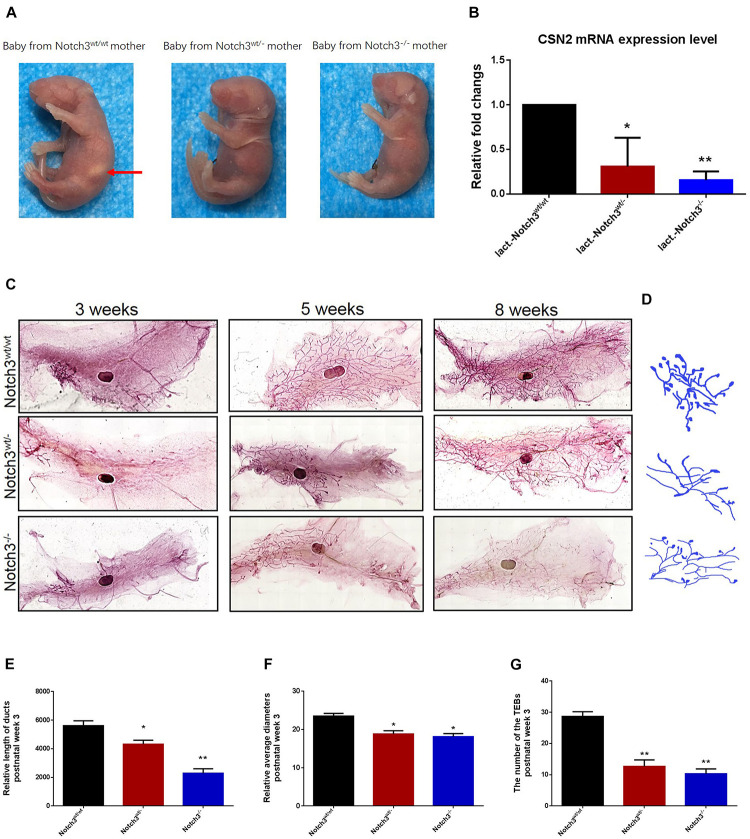
Notch3 is required for the development of mammary glands in mice. **(A)** Milk in the stomachs of the babies produced by heterozygous and homozygous Notch3 knockout mothers was not observed. **(B)** The CSN2 mRNA expression levels in mammary gland tissues at lactation stage were detected by qRT-PCR. **(C)** The fourth pair of mammary glands were harvested from 3, 5, and 8 weeks post-birth female Notch3^wt/wt^, Notch3^wt/–^, and Notch3^–/–^ FVB background mice. The mammary glands were fixed in methanol (10%), stained overnight with Carmine dye, and scanned using the Perkin Elmer Vectra Slide Analysis System. The development of mammary glands from Notch3^wt/–^ and Notch3^–/–^ mice was clearly disrupted and affected both ductal and alveolar growth. **(D)** The profiles of primary duct, secondary branching, tertiary branching, and the terminal end buds (TEBs) are extracted from whole-mount mammary gland samples. **(E)** The relative length of total ducts in mammary glands at 3 weeks of age is measured. **(F)** The mean diameter of total ducts in mammary glands at 3 weeks of age is measured. **(G)** The number of total TEB in mammary glands at 3 weeks of age is counted. Data are presented as mean ± SD of three independent experiments. The asterisks indicate statistical significance (*p < 0.05; **p < 0.01).

In view of the important phenotypes that female Notch3^wt/–^ or Notch3^–/–^ mice are unable to support live birth because of an inability to lactate (Figure 1A) as well as the decreased expression of CSN2 (β-casein), in turn, in heterozygous and homozygous Notch3 knockout mothers (Figure 1B), we speculated that Notch3 should be required for mouse mammary gland development. To verify this, we collected mouse mammary gland tissues at several key time points, such as week 3 (before puberty, *n* = 3), week 5 (puberty, *n* = 3), and week 8 (after puberty and mature, *n* = 3).

Like normal developing mammary glands, the rudimentary ductal had formed in Notch3^wt/wt^ mice, and the ducts and the branches were elongated toward the lymph node, and the terminal end buds (TEBs) were numerous at 3 weeks of age. By contrast, in Notch3^wt/–^ and Notch3^–/–^ mice, although the rudimentary epithelial anlage (the rudimental ductal tree or TEBs) had formed, a small number of ducts or very tiny TEBs were observed. At 5 weeks of age, the main ducts reached or exceeded the lymph node, and side branches with TEBs were abundant and elongated to the edge of the fat pad in Notch3^wt/wt^ mice. However, elongated ducts with only a few side branches and TEBs were found under or just above the lymph node in Notch3^wt/–^ and Notch3^–/–^ mice. Until 8 weeks after birth, the ductal system was well implanted in the fat pad with numerous side branches with a little evidence for TEBs in Notch3^wt/wt^ mice. Nevertheless, the ducts and the lobuloalveolar structure were shorter and sparser in Notch3^wt/–^ and Notch3^–/–^ mice as compared to the controls (Figure 1C).

Limited by the complex morphological structure of mammary glands at 5 and 8 weeks of age, we measured the relative length and the mean diameter of ducts and counted the number of TEBs in three genotype mice at 3 weeks of age. We first noticed that the total relative length of the primary duct of the mammary glands in either Notch3^wt/–^ or Notch3^–/–^ mice was shorter than that in age-matched control Notch3^wt/wt^ mice. The relative length of the primary ducts of the mammary glands was 5,616.02 ± 342.89, 4,374.10 ± 277.34, and 1,902.19 ± 314.21 μm in Notch3^wt/wt^, Notch3^wt/–^, and Notch3^–/–^ mice, respectively. There was a significant difference between Notch3^wt/wt^ and Notch3^wt/–^ or Notch3^–/–^ mice (Figures 1D,E). Next, we found that the relative mean diameter of the ducts in Notch3^wt/–^ and Notch3^–/–^ mice (18.81 ± 0.86 and 18.12 ± 0.82 μm) was thinner than that in Notch3^wt/wt^ mice (24.89 ± 0.70 μm). There were statistical differences between Notch3^wt/wt^ and Notch3^wt/–^ as well as between Notch3^wt/wt^ and Notch3^–/–^ mice (Figures 1D,F). Finally, we also noticed that the number of TEBs in Notch3^wt/wt^ mice (28.67 ± 1.53 (μm) was markedly more than that in Notch3wt/^–^ or Notch3^–/–^ mice (12.67 ± 2.08 and 10.33 ± 1.53 μm) (Figures 1D,G). Similar phenotypes could be observed in Notch3^wt/–^ and Notch3^–/–^ mice at 5 and 8 weeks of age. These results suggest that Notch3 is required for ductal morphogenesis during mouse mammary gland development.

### Significant Gene Expression Profile Changes Related to Notch3 Knockout

To explore the underlying mechanism involved in the failure of mammary gland development in Notch3 knockout mice, we compared the gene expression profiles of developing mammary glands derived from the three mice genotypes using RNA-Seq. In total, 16,039, 16,354, and 15,895 genes were detected at about 3 weeks of age, 16,037, 16,261, and 16,317 genes were detected at about 5 weeks of age as well as 16,130, 16,263, and 16,351 genes were detected in Notch3^wt/wt^ (*n* = 3), Notch3^wt/–^ (*n* = 3), and Notch3^–/–^ mice (*n* = 3) at about 8 weeks of age, respectively.

**FIGURE 2:**
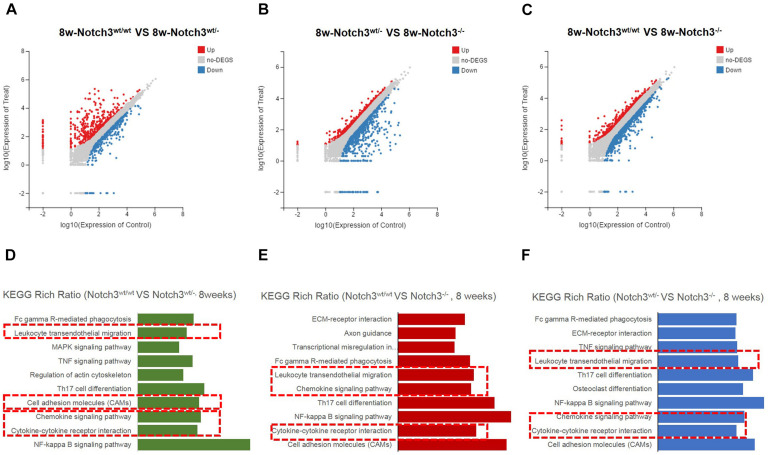
RNA-Seq and Kyoto Encyclopedia of Genes and Genomes (KEGG) enrichment analyses were performed. **(A–C)** Scatter plots of differentially expressed gene mRNAs in mammary glands with three different genotypes at 8 weeks of age, respectively. **(D–F)** KEGG pathway enrichment is used to identify the chemokine pathway modulated by Notch3.

**FIGURE 3:**
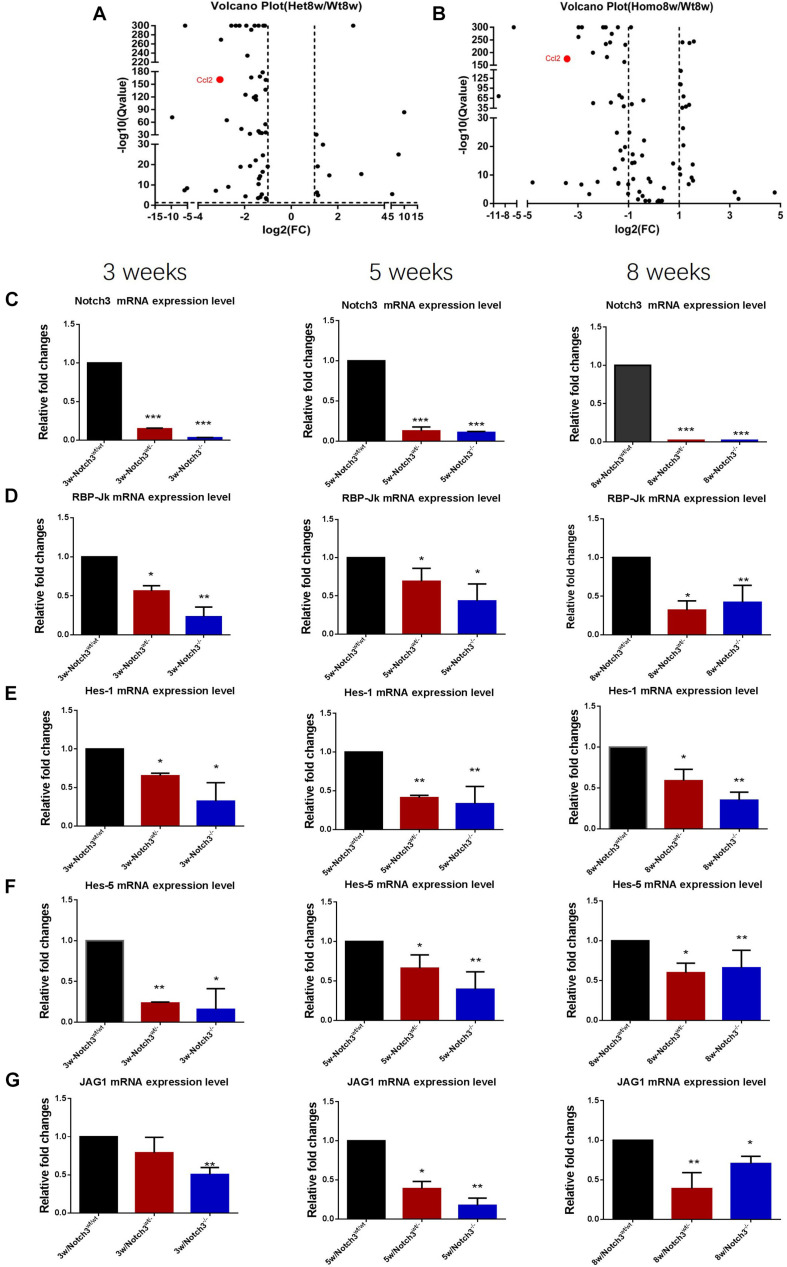
RNA-Seq analysis reveals that Notch3 knockout suppresses CCL2 expression. **(A,B)** Volcano plots showing the log2 fold changes and the –log10 *Q*-values in transcript cluster expression in the chemokine signaling pathway of the mammary gland tissue transcriptome profiles Notch3 knockout mice compared to the wild-type controls. CCL2 expression is significantly reduced in the mammary gland tissues of Notch3 knockout mice. **(C–G)** The expression levels of RBP-Jκ, Notch3 receptor, its ligands Jagged1, and the canonical Notch target genes of the Hes/Hey family (Hes1, Hes5) at age of 3, 5, and 8 weeks postnatal were examined by real-time qRT-PCR. Data are presented as mean ± SD of three independent experiments. The asterisks indicate statistical significance (*p < 0.05; **p < 0.01; ***p < 0.001).

Of these detected differentially expressed genes (DEGs) at about 8 weeks of age, 483 genes were upregulated and 332 genes were downregulated in Notch3^wt/wt^ *versus* Notch3^wt/–^, 830 genes were upregulated and 731 genes were downregulated in Notch3^wt/–^ *versus* Notch3^–/–^, and 862 genes were upregulated and 742 genes were downregulated in Notch3^wt/wt^ *versus* Notch3^–/–^ mammary tissue samples (Figures 2A–C). The Kyoto Encyclopedia of Genes and Genomes (KEGG) pathway analysis was carried out on all screened DEGs, and the top 10 enriched pathways are shown in Figures 2D–F. Upon comparison of the candidate pathways, we found that chemokine signaling, Th17 cell differentiation, leukocyte trans-endothelial migration, and cytokine–cytokine receptor interaction pathways were shared between the Notch3^wt/wt^ *versus* Notch3^wt/–^ and Notch3^wt/wt^ *versus* Notch3^–/–^ tissue samples. The volcano plots show the up-regulated and the down-regulated genes (DEGs) in the chemokine signaling pathway (Figures 3A,B), in which the decreased CCL2 expression significantly attracted our attention. The results point out that Notch3 knockout significantly changes the gene expression profile of mice, especially the chemokine signaling pathway.

### The Classical Notch Signaling Pathway Was Inactivated in Notch3 Knockout Mice

To verify the RNA-seq results and explore which ligand or downstream molecules in the Notch signaling pathway were mainly influenced after Notch3 knockout, the expression levels of RBP-Jκ, Notch3 receptor, its ligands (Jagged1/2 and DLL 1, 3, and 4), and the canonical Notch target genes of the Hes/Hey family (Hes1, Hes5, Hey1, and Hey2) at postnatal 3, 5, and 8 weeks of age were examined by real-time qRT-PCR. The results revealed that the expression of Notch3 and RBP-Jκ was significantly downregulated, accompanied by Notch3 knockout. Of the five possible Notch ligands, only JAG1 was found to be significantly downregulated in Notch3 knockout mammary glands tissues. The expression of other ligands was undetected (data not shown). Of the possible targets within the Hes/Hey family, Hes1 and Hes5 were identified (Figures 3C–G). These data indicate that Notch3 knockout inactivated the classical Notch signaling pathway, and N3ICD preferentially regulates the expression of Hes1 and Hes5 by interacting with JAG1.

### CCL2 Expression Was Downregulated in Mammary Glands of Notch3 Knockout Mice

**FIGURE 4:**
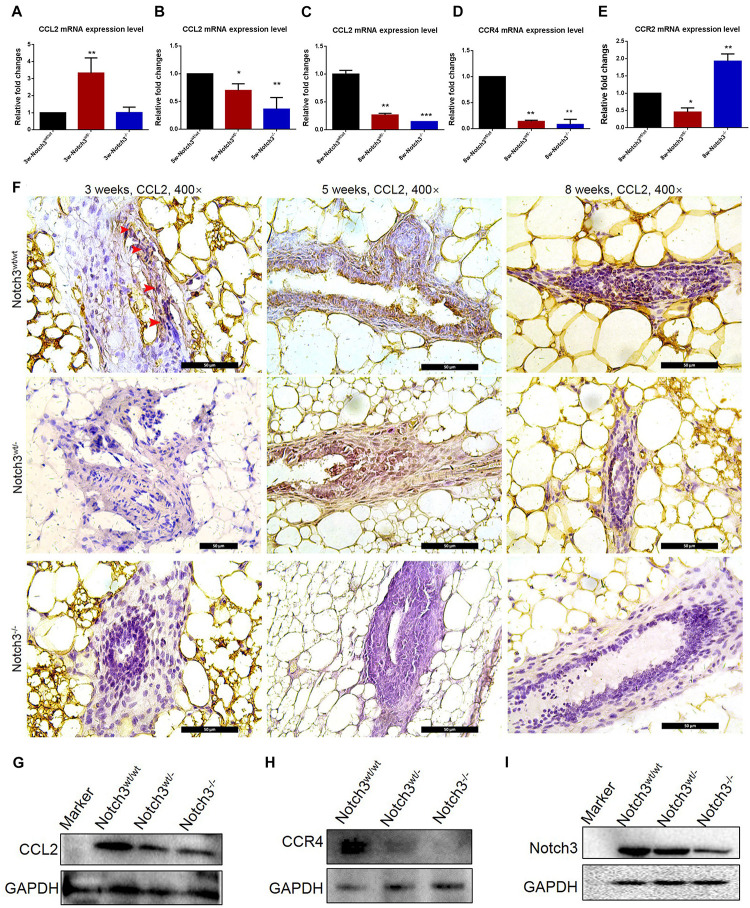
CCL2 expression decreases in mammary gland tissues of Notch3 knockout mice. **(A–C)** The expression levels of CCL2 at age of 3, 5, and 8 weeks postnatal were examined by real-time qRT-PCR. **(D,E)** The expression levels of CCL2 receptor (CCR2 and CCR4) at 8 weeks of age were examined by real-time qRT-PCR. **(F)** Immunohistochemistry of CCL2 using an antibody specific to CCL2 in Notch3^wt/wt^, Notch3^wt/–^, and Notch3^– /–^ mammary gland tissues at three time points (10% methanol-fixed, paraffin sections; ×20 magnification). **(G–I)** The expression levels of Notch3, CCL2, and CCR4 of mammary glands at 8 weeks were determined by western blotting. GAPDH was used as internal control. The asterisks indicate statistical significance (*p < 0.05; **p < 0.01; ***p < 0.001).

**FIGURE 5:**
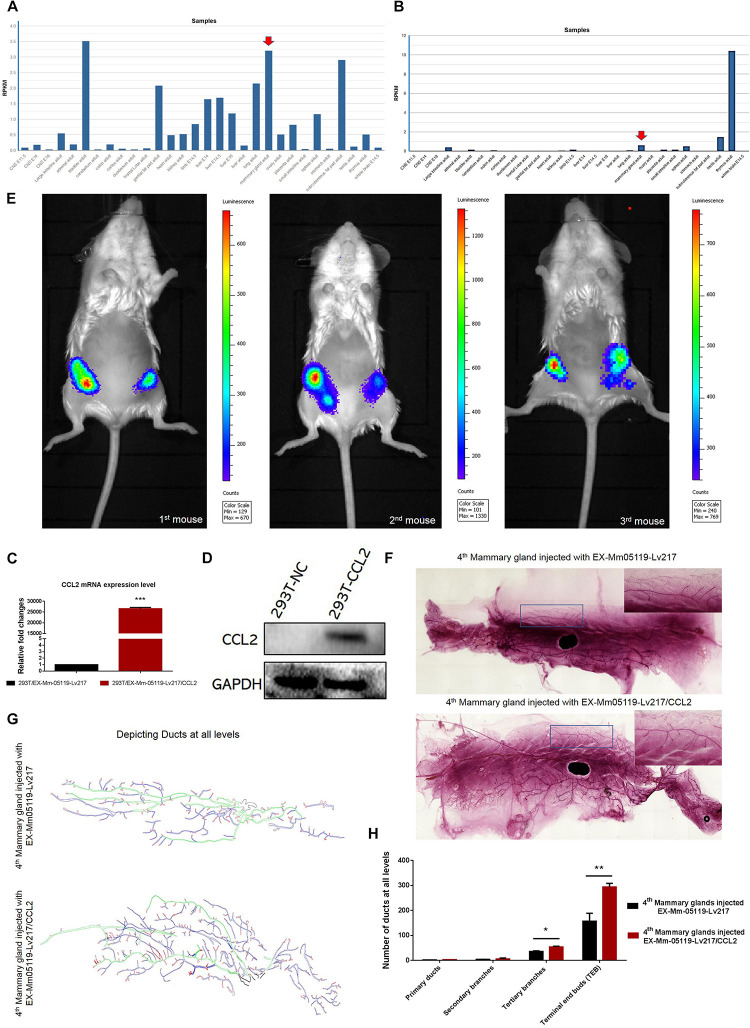
CCL2 and its receptor CCR4 may be important for mouse mammary gland development. Intraductal injection of CCL2 ectopic overexpression lentivirus can partially rescue the failure of mammary gland development resulting from Notch3 knockout. **(A,B)** Mouse ENCODE transcriptome data reveal that the CCL2 and CCR4 expression levels in the mammary gland fat pad respectively rank fifth and third in a total of 30 mouse embryonic and adult tissue samples. **(C,D)** Determining the overexpression efficiency of CCL2 by qRT-PCR and western blotting. **(E)** Representative images of luminescence in mice showing the effect of CCL2 on rescued mammary gland development by Luc2 activity measurement. For all experiments, three mice were used in this experiment. The experimental groups consisted of left mammary glands (ventral view) of three mice injected with CCL2 overexpression lentivirus. The control groups consisted of right mammary glands (ventral view) of three mice injected with empty vector lentivirus with the same concentration as the CCL2 overexpression lentivirus. **(F)** The whole mount the 4th pair of mammary glands injected with EX-Mm05119-Lv217 (upper) or EX-Mm05119-Lv217/CCL2 (bottom) lentivirus were fixed in 10% methanol, stained overnight with carmine alum dye, and scanned using the Perkin Elmer Vectra Slide Analysis System. **(G)** The pattern diagram shows the depicted primary duct (green color), second branching (blue color), tertiary branching, and terminal end buds (TEBs) (red color) of mammary glands. Control mammary gland (upper) and experimental mammary gland (bottom). The enlarged views of the areas indicated by the rectangles. **(H)** Quantitation of the numbers of the primary duct, second branches, tertiary branches, and TEBs of mammary glands of Notch3 knockout mice after intraductal injection of EX-Mm05119-Lv217 or EX-Mm05119-Lv217/CCL2 lentivirus. The asterisks indicate statistical significance: *p < 0.05, **p < 0.01, and ***p < 0.001 (Student’s *t*-test) as compared to control cells. Data are presented as mean ± SD (*n* = 3).

To evaluate whether Notch3 knockout affects CCL2 expression in mammary gland tissues, real-time qRT-PCR was performed. The RT-PCR results showed that CCL2 expression was reduced almost at all stages besides Notch3 heterozygote at postnatal 3 weeks of age (Figures 4A–C). In addition, we also detected the mRNA expression levels of two CCL2 receptors, such as CCR2 and CCR4, in mammary gland tissues with three different genotypes at 8 weeks of age. The results showed that, although there were no rules to follow for CCR2 expression, decrease in Notch3^wt/–^, and increase in Notch3^–/–^, CCR4 expression was, however, significantly downregulated either in Notch3^wt/–^ or in Notch3^–/–^ mammary gland tissues as compared with that in Notch3^wt/wt^ mice, suggesting that CCR4 was selectively decreased (Figures 4D,E). Furthermore, anti-CCL2 immunohistochemistry staining was performed on paraffin sections of mammary gland tissue representing the three genotypes. The immunohistochemistry staining revealed that the higher intensity staining was mainly located in the cytoplasm of epithelial cells and some stromal cells in the Notch3^wt/wt^ mammary gland tissues at 3, 5, and 8 weeks of age (Figure 4F, top panel). By contrast, CCL2 immunohistochemistry staining revealed weak positive signals or negative signals in Notch3^wt/–^ or in Notch3^–/–^ mammary gland tissues in the three age groups (Figure 4F, middle and bottom panels). The expression levels of CCL2 and CCR4 in the mammary gland tissues at 8 weeks were determined by western blot. The results showed that the expression of both CCL2 and CCR4 significantly reduced along with Notch3 knockdown (Figures 4G–I). This result is also supported by data of the RNA-Seq dataset which were obtained from the ENCODE project, an assortment of 30 samples of mouse tissue from eight different embryos, covering several stages of embryo development, as well as 22 adult tissues of mouse^1^ (Parkhomchuk et al., 2009; Jiang et al., 2011). The Mouse ENCODE transcriptome data reveal that the CCL2 expression level in mammary gland fat pad ranks the second in a total of 30 mouse embryonic and adult tissue samples (Figure 5A). Additionally, the receptor CCR4 presented the third highest mRNA expression level in a total of 30 mouse embryonic and adult tissue samples (Figure 5B). Overall, the results reveal that CCL2 and its receptor CCR4 are particularly important for mouse mammary gland development and that Notch3 knockout leads to the reduced expression of CCL2 and CCR4 receptor.

### Overexpressing CCL2 *in vivo* via Intraductally Injecting Can Partially Rescue the Mouse Mammary Glands Deficiency Resulting From Notch3 Knockout

To explore whether CCL2 represents a feasible target for developing mammary glands in Notch3 knockout mice, a rescue experiment was performed. First, we constructed a CCL2 expression lentivirus vector, EX-Mm05119-Lv217/CCL2, containing the firefly luc2 gene. Plasmids were transiently transfected into 293T cells to verify the overexpression efficiency. Both real-time qRT-PCR and western blotting showed that the expression level of CCL2 in 293T cells transfected with EX-Mm05119-Lv217/CCL2 was sharply raised as compared to the control cells (Figures 5C,D). In addition, we also validated the expression of firefly luciferase Luc2 gene; the result is shown in the supporting information (Supplementary Figure S2). These data illustrate that EX-Mm05119-Lv217/CCL2 vector was constructed successfully.

Next, CCL2-overexpressing lentivirus that carried Luc2 tag were delivered by intraductal injection through the nipple. To ensure accurate injection, we firstly injected trypan blue to test the injection volume in the mouse mammary glands (shown in the Supplementary Figure S3). After the CCL2-overexpressing lentivirus was delivered, the mice were subjected to non-invasive fluorescent *in vivo* imaging using PerkinElmer’s IVIS imaging platform. The results showed that a bioluminescent reporter of gene activity was observed within mammary epithelial cells, and no fluorescent particles were observed in the other organs. In detail, the experimental left inguinal (ventral view) presented a wide distribution of potent luminescent signal, which was equal to the localization of the mammary tissue. Conversely, the control right inguinal (ventral view) showed foci of luminescent signals (Figure 5E). On average, total luminescence readout from the left inguinal significantly exceeded that from the control right inguinal by 3.945-fold (at 10-week time point, *n* = 3). At the endpoint of the experiments, the mammary glands were isolated, and whole-mount preparation as well as carmine alum staining was carried out. The number of primary, secondary, and tertiary ducts and TEBs was counted. The results showed no obvious changes in the numbers of primary and secondary ducts between experimental mammary glands and control mammary glands. However, the numbers of tertiary ducts and TEBs in the experimental mammary glands (Figure 5F—bottom, Figure 5G—bottom, and Figure 5H) were more than that in the control mammary glands (*n* = 3) (Figure 5F—upper, Figure 5G—upper, and Figure 5H). We can conclude that the delivery of CCL2 overexpression lentivirus by intraductal injection can partly rescue the structures of the tertiary ducts and TEBs in Notch3 heterozygous or homozygous mice.

### Breast Cancer Patients With High CCL2 Expression Are Closely Associated With More Malignant Behaviors

The development or regeneration of organ/tissue and carcinogenesis are likely closely related processes. We therefore used Gene Expression-Based Outcome for Breast Cancer Online dataset to analyze and compare the CCL2 expression level among various breast cancer molecular subtypes according to HU or PAM50 subtyping, namely, basal, HER2 positive, luminal A, luminal B, normal-like, and unclassified breast cancer^2^. Of these six subtypes, we observed that both luminal A and luminal B present lower CCL2 expression levels, whereas basal-like, HER2 positive, normal-like, and unclassified breast cancer exhibit higher CCL2 expression levels (*p* < 0.00001, Supplementary Figure S1A—upper row). Based on ER status, we analyzed and compared the CCL2 expression levels and found that there is significantly higher CCL2 mRNA expression in ER-negative than in ER-positive breast cancer (*p* < 0.00001; Supplementary Figure S1A—lower-left). Additionally, a statistical analysis comparing the expression levels according to tumor grade showed that there are significantly higher CCL2 mRNA expression levels in grade III tumors than in grade II or I tumors (*P* = 0.00819, Supplementary Figure S1A—lower-right). Importantly, a survival analysis from cBioPortal dataset demonstrates that the higher CCL2 expression levels are significantly correlated with the poor survival rate of breast cancer patients (*p* = 0.0202) (Supplementary Figure S1B). Those patients whose tumors had higher levels of CCL2 expression had worse distant metastasis-free survival (*p* = 0.00085) (Supplementary Figure S1C). Altogether these data demonstrated that CCL2 may play pivotal roles during the development of mammary glands and breast cancer progression.

### Notch3 Knockdown Inhibits the Proliferation and the Migration of 4T1 Cells via CCL2/CCR4 Axis

**FIGURE 6:**
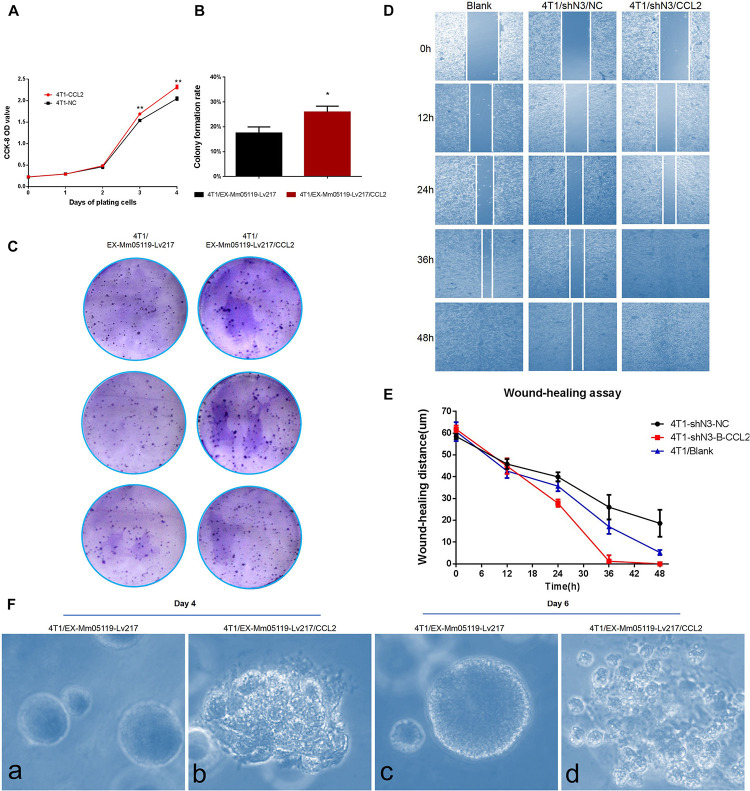
CCL2 overexpression can promote proliferation and malignant acinar structure formation of 4T1 cells. **(A)** Transient overexpression of CCL2 promotes 4T1 cell growth *in vitro* (*p* < 0.01). EX-Mm05119-Lv217/CCL2 plasmids are transfected into the 4T1 cells. EX-Mm05119-Lv217 plasmid is used as control. The cells’ growth absorbance is measured using CCK-8 kit at the indicated time points. **(B,C)** Representative colony formation assays in monolayer culture to assess the tumorigenesis function of 4T1 cells treated with CCL2 overexpression. **(D,E)** Wound healing assays are performed in 4T1 blank and 4T1/shN3/NC and 4T1/shN3/CCL2 cells, and wound healing distance at different time points are shown. **(Fa,c)** Acinar formation assay. The acinar structures of 4T1 cells at days 4 and 6 after transfection with EX-Mm05119-Lv217. **(Fb,d)** The acinar structures of 4T1 cells transfected with EX-Mm05119-Lv217/CCL2, which was cultured in Matrigel for 4 and 6 days, ×10. Data are presented as mean ± SD of three independent experiments, and asterisks indicate statistical significance (*p < 0.05; **p < 0.01).

We next tested the effect of CCL2 on the proliferation of mouse triple negative breast cancer 4T1 cells by CCK8 assay and colony formation assay. The CCK8 analysis showed that overexpressing CCL2 could significantly enhance the proliferation of 4T1 cells after 3 days of plating the cells as compared with empty vector-transfected 4T1 cells (*P* < 0.01) (Figure 6A).

The result was further confirmed by a following colony formation assay, which showed that overexpressing CCL2 markedly increased the number of colony formation of 4T1 cells (25.93 ± 2.35%) compared to control cells (17.50 ± 2.45%, *p* < 0.05) (Figures 6B,C).

To further validate that Notch3 plays roles *via* the CCL2/CCR4 axis, a functional rescue experiment was conducted by knocking down Notch3 combined with overexpressing CCL2. 4T1 cell migration detected by wound healing (or gap closure) assay revealed that Notch3 knockdown significantly retarded wound closure. By contrast, the gap was filled with control 4T1 cells at 48 h. Furthermore, the slow migration of 4T1 cells caused by Notch3 knockdown could be restored by overexpressing CCL2, which made the gap close by about 75% after 24 h and completely fill at 36 h (Figures 6D,E). These data indicate that ectopic CCL2 overexpression can partially compensate for the migration deficiency of 4T1 cells caused by Notch3 knockdown.

It is well known that normal mammary gland epithelial cells have several distinguishing histological features, including a polarized morphology, specialized cell–cell contacts, attachment to an underlying basement membrane, and organized acinar structures. We asked whether the ectopic overexpression of CCL2 in 4T1 cells can affect the organized acinar structures. We therefore used Matrigel 3D culture which can simulate an approximate reconstruction of the tissue architecture, such as duct, lobuloalveolar, and basement membrane. The results showed that the experimental 4T1 cells transfected with CCL2 overexpression vectors began to proliferate quickly and exhibited several oncogenic phenomena at day 4, such as invasive ductal structures extending outward, disorganized architectural morphology in the 3D Matrigel, as well as increased number of abnormal grape-like tumor structures (or 3D acinar structures) (Figure 6Fb). On the contrary, the 4T1 cells transfected with empty vector began to proliferate on reconstituted basement membrane and make well-organized 3D spherical architectures (Figures 6Fa,c). By the 6th day, the 4T1 cell-overexpressing CCL2 seemed to form more abnormal grape-like tumor structures (Figure 6Fd). These data emphasize that the overexpression of CCL2 can morphologically promote more invasive 3D acinar structure formation. Collectively, CCL2 overexpression can promote the proliferation and the migration of 4T1 cells and can rescue slow proliferation and migration caused by Notch3 knocking down.

### Notch3 Regulates CCL2 Expression at the Transcriptional Level

**FIGURE 7:**
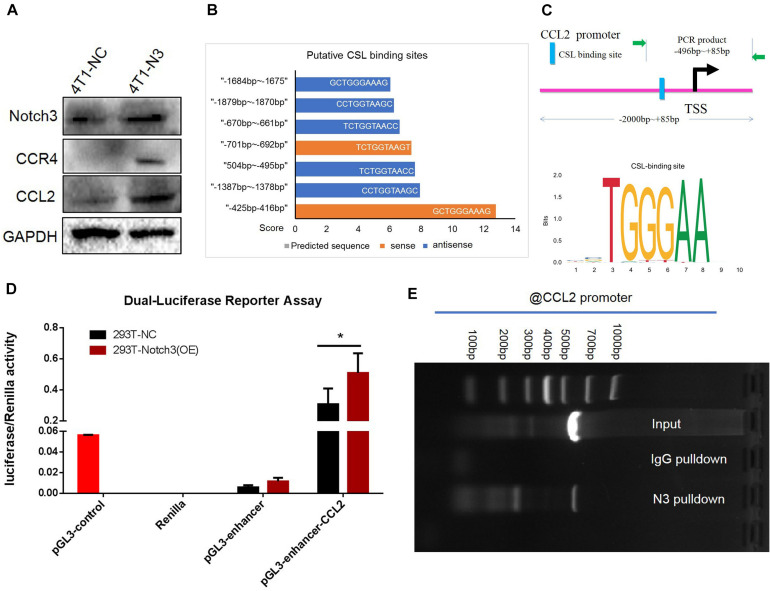
Chromatin immunoprecipitation (ChIP) and luciferase assays confirm that CCL2 is regulated by Notch3 at the transcriptional level. **(A)** Western blot verified that ectopic Notch3 overexpression can upregulate the expression of CCL2. **(B)** The predicted RBP-Jκ binding sites (GCTGGGAAAG, in the -strand) in the CCL2 promoter and their scores by Jaspar online software. The scale in the *X*-axis represents score, orange color represents the sense sequence of DNA chain, and blue color represents the antisense sequence of DNA chain. **(C)** The pattern diagram shows the sequence and the location in CCL2 promoter of Notch3/RBP-Jκ binding to the RBP-Jκ binding site. **(D)** The fragment of CCL2 promoter containing RBP-Jκ binding site (–496 to +85 bp) was inserted into the luciferase reporter vectors by two restricted endonucleases [Sac I (CGAGCTCG) and Sma I (TCCCCCGGGGGA)]. 293T-Notch3 overexpression cells and 293T-NC control cells were co-transfected with pGL3-enhancer CCL2-promoter reporter plasmid, pGL3-enhancer vector, or pGL3-control vector with Renilla luciferase reporter vector, respectively. Luciferase activity was normalized to that of Renilla. **(E)** The ChIP assay used normal IgG (IgG) or anti-Notch3 antibody to determine whether Notch3 can bind the RBP-Jκ binding site in the CCL2 promoter in C2C12 cells (induced in a medium containing 1% horse serum for 2 days). After ChIP, PCR reveals that the Notch3/RBP-Jκ complex binds to the RBP-Jκ binding site in the antisense orientation. *p < 0.05 (Student’s *t*-test) as compared to the control cells. Data are presented as mean ± SD (*n* = 3).

To verify the positive correlation between Notch3 and CCL2 in mouse mammary gland tissues, we also ectopically expressed Notch3 in mouse 4T1 cells and tested the CCL2 expression level. The western blot assay confirmed that Notch3 overexpression led to a raised expression of CCL2 (Figure 7A). In view of the fact that Notch3 is an important transcription factor, we hypothesized that transcription factor Notch3 may directly regulate CCL2 gene expression. Analyzing the sequence of promoter and first non-encoding exon of CCL2 (−2,000 to +85 bp) by an online tool^3^, we noticed that a canonical RBP-Jκ binding site at the position between −425 and −416 bp on the sense strand showed a high score (12.75), suggesting that Notch3 might act as an upstream inducer of CCL2, most likely in a direct manner (Figures 7B,C). The luciferase reporter vector that included the region from −496 to +85 bp of the CCL2 gene (pGL3-enhancer CCL2-promoter reporter plasmid) was constructed. A dual luciferase reporter assay was used to investigate CCL2 promoter activity in the established Notch3 overexpressing 293T cells and control 293T-NC cells by transient co-transfection of pGL3-enhancer CCL2-promoter reporter plasmid, pGL3-enhancer vector, or pGL3-control vector with Renilla luciferase reporter vector. CCL2 promoter activity increased 2.12- to 35.48-fold in Notch3 overexpressing 293T cells when compared with 293T cells transfected with an empty vector, suggesting that Notch3 effectively elicits CCL2 expression in a classical Notch signaling pathway activation pattern. By contrast, the CCL2 promoter activity was not significantly affected in those control 293T-NC cells (Figure 7D; *p* < 0.05). We further conducted ChIP assay with primers covering the RBP-Jκ binding sites in the CCL2 promoter region in Notch3-overexpressing C2C12 cells. The Notch3 antibody was used to identify the Notch3/RBP-Jκ binding site on the CCL2 promoter; non-specific IgG (IgG) was used as a negative control, and input was used as a positive control. PCR was used to examine the RBP-Jκ occupancy of the putative loci. As expected, binding was observed (Figure 7E) in Notch3 antibody pull-down group. These data suggest that Notch3 activates CCL2 expression by binding to the RBP-Jκ-binding site of the CCL2 promoter. To sum up, these results demonstrate that Notch3 directly activates CCL2 transcription by specifically binding to the promoter region of the CCL2 gene.

## Discussion

Mammals have four Notch paralogs (Notch1–4); the presence of different receptors will also influence the signaling outcomes (Bellavia et al., 2008; Penton et al., 2012; Kopan et al., 2014). In this study, we investigated the influence of Notch3 on mammary gland development by using Notch3 knockout mice as well as the effect of Notch3 on the biology behaviors of the murine breast cancer cell 4T1 by overexpressing or knocking down Notch3. We present *in vitro* and *in vivo* evidence and the molecular mechanism by which Notch3 plays an important role in promoting mammary gland development and suppressing breast cancer cell growth, invasion, and migration.

Interestingly, in this study, what firstly caught our attention was that Notch3-deficient mothers would not like to raise their pups, leading to the fact that most of the newborn pups died soon after birth. Secondly, we found that the mRNA expression levels of CSN2 (β-casein) decreased in those mature mammary gland tissues derived from heterozygous and homozygous Notch3 knockout mice as compared with wild mice. Thirdly, Notch3 deficiency delays mouse mammary gland development and branching morphogenesis after evaluating gland architecture at three main development time points, namely, juvenile, pubertal, and mature virgin (Topper and Freeman, 1980). Obviously, these observations indicated that Notch3 appears to be a master regulator that is required for ductal and lobuloalveolar morphogenesis during mammary gland development. A published study has shown that Notch3 marks clonogenic mammary luminal progenitor cells *in vivo* (Lafkas et al., 2013).

Then, we wanted to know how Notch3 knockout affects mammary gland development. The transcriptome expression profiles of mammary gland tissues with three different Notch3 genotypes were analyzed by RNA-Seq. Based on KEGG and GO analysis of the DEGs, chemokine signaling, Th17 cell differentiation, leukocyte transendothelial migration as well as cytokine–cytokine receptor interaction pathways were in the top 10 enriched pathways. Of these DEGs made up above pathways, CCL2 was involved in multiple pathways, whose expression level was significantly suppressed either in Notch3 heterozygous or homozygous mammary gland tissues, suggesting that CCL2 is likely to be closely related to Notch3 and that it may be a key signaling molecule during mammary gland development. Therefore, we chose to focus our further analysis on CCL2.

CCL2, a 13-kDa CC family of chemokines and also known as monocyte chemotactic protein-1 (MCP-1/CCL2), is produced mainly by monocytes/macrophages but is also expressed by endothelial, fibroblasts, epithelial, smooth muscle, mesangial, astrocytic, and microglial cells. Recently, Ling et al. (2019) showed that oral squamous carcinoma cells can express and release CCL2 to effectively induce epithelial–mesenchymal transition. Moreover, there is a growing body of evidence that CCL2 has the ability to recruit monocytes and macrophages during development, inflammation, and tumorigenesis (Ferreira et al., 2008). Those recruited macrophages and eosinophils persist in surrounding the TEBs through development but disappear as soon as the TEBs turn into terminal end ducts (Coussens and Pollard, 2011). The cap cells at the TEBs that are thought to be a reservoir for regenerative mammary stem cells have a higher ability to form an entire ductal tree (Paine and Lewis, 2017). Probably the expression and the release of CCL2 by mammary gland epithelium contribute to the TEB elongation, invasion, and development. In this study, to evaluate whether Notch3 knockout affects the CCL2 expression in mammary gland tissues, we performed real-time qRT-PCR. Interestingly, the RT-PCR results showed that CCL2 expression was reduced almost at all time points besides Notch3 heterozygote at postnatal 3 weeks of age. We speculated that the possible reason should be that CCL2 compensatively and transiently increased in Notch3 heterozygous loss in mammary glands at 3 weeks. Indeed the expression level of CCL2 presented a declined trend in Notch3 homozygous loss in mammary glands at 3 weeks. Importantly, we clearly observed that the expression level of CCL2 significantly downregulated in Notch3 heterozygous mammary gland tissue with development, such as at 5 and 8 weeks. The long-term absence of CCL2 leads to breast development defects. Obviously, the delivery of overexpressing-CCL2 lentivirus particles increased the number of TEBs, suggesting that CCL2 overexpression could partially rescue the developmental deficiency of mammary glands caused by Notch3 knockout. Our current conclusion is indirectly supported by others’ reports. CCL2 induced Notch1 expression and the cancer stem cell features in breast cancer cells, constituting a “cancer–stroma–cancer” signaling circuit (Tsuyada et al., 2012).

In line with mammary gland development, we also found that ectopic CCL2 overexpression can partially reverse the decreased proliferation and the migration of 4T1 cells caused by Notch3 knockdown and can promote invasive acinar formation. Our results were supported by published studies. Zhang et al. (2010) showed that the elevated expression of CCL2 positively correlates with the increased tumor-associated macrophages numbers in several human tumors. CCL2-treated neural stem cells showed a significantly increased capacity for self-renewal, proliferation, and neuronal differentiation (Hong et al., 2015). Loss of CCL2 significantly inhibited tumorigenesis (Tsuyada et al., 2012). These evidences suggest that CCL2 is an important molecule during both mammary gland development and breast cancer progression.

However, it is unclear how Notch3 influenced mammary gland development *via* CCL2 expression. Given that Notch3 is a transcriptional factor, we suspected that Notch3 may participate in mammary gland development *via* transcriptionally regulating chemokine CCL2 for a number of reasons: (1) ectopic Notch3 overexpression raised the expression level of CCL2 in mouse 4T1 breast cancer cells; (2) a putative RBP-Jκ binding site was observed in the promoter of CCL2; (3) the results from a luciferase activity assay revealed that Notch3 overexpression significantly increased luciferase activity; and (4) the ChIP experiment showed that Notch3 apparently bound to the RBP-Jκ binding site in the promoter of CCL2. We can conclude that Notch3 may be a master gene for building a nice microenvironment for mammary development by directly and transcriptionally regulating CCL2 expression. The study from other people showed that Notch activation that is associated with basal-like breast cancer normally directs tissue patterning, suggesting that it may shape the tumor microenvironment (Shen et al., 2017). Based on these facts, our further study may focus on whether Notch3 play vital roles in a cancerous microenvironment *via* regulating CCL2 signaling pathway.

Besides acting through CCR2 (Sun et al., 2017), CCL2 was found to also activate CCR4 (Ishida and Ueda, 2006; Frossard et al., 2011) that is responsible for the more potent chemotaxis of mouse macrophages (Liu et al., 2013). Furthermore, macrophages are required for ductal morphogenesis in postnatal mammary gland development (Gouon-Evans et al., 2000). In addition, in analogy with immune cells, tumor dissemination requires chemokine receptors. To date, it has been reported that the expression of CXCR4, CCR7, and CCR10 was associated with breast cancer metastases (Muller et al., 2001). Specifically, Olkhanud et al. (2009) reported that a proportion of tumor cells may express CCR4 and migrate to the lungs the same way as the CCR4+ immune cells.

It is worth mentioning that the *in vivo* rescue experiment carried out through intraductal injection in this study was frequently compromised. We believed that the possible reasons for this are as follows: (1) the nipples and the primary duct are usually dysplasia either in Notch3^wt/–^ or Notch3^–/–^ mice; it is hard to see the nipples from postnatal 8-week mice even under a microscope (Supplementary Figure S4); (2) the duct blockage in some mice resulted in failed or compromised CCL2 lentivirus delivery because non-lactating virgin females were used in this study; (3) too fast and too much injection led to duct rupture; and (4) incorrect technique. To overcome these difficulties, we basically invited experienced researchers in our laboratory to perform this study.

In this study, an interesting finding that N3ICD/RBP-Jκ preferentially binds to the RBP-Jκ binding site in the antisense orientation (Liang et al., 2002; Zhang et al., 2016) was verified again. The nearest RBP-Jκ binding site to the transcription start site with the highest score predicated is still on the antisense strand (5′-TTCCCAG-3′; −425 to −416 bp), which was further verified by luciferase activity assay and ChIP in this study. The phenomenon that N3ICD binds to RBP-Jκ binding sites in the antisense orientation may lead to a higher probability of Notch activation, which is probably one of the important reasons why Notch3 acts differently from other members in the same family, such as Notch1, 2, and 4.

Finally, breast cancer is the most common cancer in women worldwide and remains a leading cause of cancer death. It is axiomatic that the study on the fundamental processes of mammary gland development can provide us great promise to hold therapeutic interventions for breast cancer patients. Our findings illustrated that Notch3 signaling activation is necessary for the development of the mammary gland, but its hyperactivation closely relates to breast cancer progression, and that Notch3-regulating CCL2/CCR4 axis should be the target for breast cancer therapy.

## Data Availability Statement

The raw data supporting the conclusions of this article will be made available by the authors, without undue reservation.

## Ethics Statement

The animal study was reviewed and approved by the ethics committee of Shantou University.

## Author Contributions

YL contributed to the study’s conception and design, the development of methodology, writing, review, and/or revision of the manuscript, and provided administrative, study, and technical supervision. WX, JT, YG, and SC contributed to the acquisition of data. WX, JT, and YL contributed to the analysis and interpretation of data (*e*.*g*., statistical analysis, biostatistics, computational analysis), Kaplan–Meier plotter analysis, and preparation of paraffin sections. ZL prepared the supporting material and kept the transgenic mice. All authors contributed to the article and approved the submitted version.

## Conflict of Interest

The authors declare that the research was conducted in the absence of any commercial or financial relationships that could be construed as a potential conflict of interest.

## Footnotes

1 https://www.ncbi.nlm.nih.gov/gene?LinkName=nuccore_gene&from_uid=141803162#top

2 http://co.bmc.lu.se/gobo

3 http://jaspar.genereg.net/

## Abbreviations

Notch3: Notch family receptor member 3
CCL2: C-C motif chemokine ligand 2
CCR2: C-C chemokine receptor type 2
CCR4: C-C chemokine receptor type 4
DEGs: differentially expressed genes
KEGG analysis: Kyoto Encyclopedia of Genes and Genomes analysis
GO: gene ontology.
