# Supplementary material for: Notch3 Knockout Suppresses Mouse Mammary Gland Development and Inhibits the Proliferation of 4T1 Murine Mammary Carcinoma Cells via CCL2/CCR4 Axis
Source: Front Cell Dev Biol. 2020 Nov 17;8:594372. doi: 10.3389/fcell.2020.594372 (PMC7685216; doi:10.3389/fcell.2020.594372)
Supplement: Supplementary file 1 [file Table_1.docx]

**Supplementary Table 1. Primers and interference sequences used in this study**

| Gene | Forward primer | Reverse primer | Purpose |
| --- | --- | --- | --- |
| CCL2 promoter | 5’- CGAGCTCGACCAAGTGGAGAGAATG -3’ | 5’- TCCCCCGGGGGATGGTGGTGGAGGA -3’ | Luciferase Repot assay |
| Notch3-wt | 5’-CCATGAGGATGCTATCTGTGAC -3’ | 5’-CACATTGGCACAAGAATGAGCC -3’ | Genotyping |
| Notch3-mut  CSN2 | 5’-TCGCCTTCTATCGCCTTCTTG -3’  5’-GGCACAGGTTGTTCAGGCTT -3’ | 5’-GGTACTGAGAACCAAACTCAG -3’  5’-AAGGAAGGGTGCTACTTGCTG-3’ | Genotyping  RT-PCR |
| CCL2  CCR2  CCR4  Notch3  18s  RBP-Jk  Hes1  Hes5  Hey1  Hey2  JAG1  JAG2  DLL1  DDL3  DLL4  CCL2 promoter | 5’-TTAAAAACCTGGATCGGAACCAA -3’  5’-ATCCACGGCATACTATCAACATC -3’  5’-GGAAGGTATCAAGGCATTTGGG -3’  5’-CGCTATGCTAGAGCGGATGC -3’  5’-AACCCGTTGAACCCCATT -3’  5’-CGGCCTCCACCTAAACGAC -3’  5’-TCAACACGACACCGGATAAAC -3’  5’-GGGTTGTTCTGTGTTTGCATTTA -3’  5’- ATCTGCTAAGCTAGAAAAAGCCG -3’  5’- AAGGCGTCGGGATCGGATAA -3’  5’-GTCCATGCAGAACGTGAACG -3’  5’- TGGGACTGGGACAACGATAC -3’  5’-CAGGACCTTCTTTCGCGTATG -3’  5’-CTGGTGTCTTCGAGCTACAAAT -3’  5’-TTCCAGGCAACCTTCTCCGA -3’  5’- CGAGCTCGACCAAGTGGAGAGAATG -3’ | 5’-GCATTAGCTTCAGATTTACGGGT -3’  5’-CAAGGCTCACCATCATCGTAG -3’  5’-GTACACGTCCGTCATGGACTT -3’  5’-AGTGGAGCGGTTCCTGATGA -3’  5’-CCATCCAATCGGTAGTAGCG -3’  5’-TCCATCCACTGCCCATAAGAT -3’  5’-GCCGCGAGCTATCTTTCTTCA -3’  5’-GAAGAAAGTCCTCTACAGGTTGG -3’  5’- GTGCGCGTCAAAGTAACCT -3’  5’- AGAGCGTGTGCGTCAAAGTAG -3’  5’-GCGGGACTGATACTCCTTGA -3’  5’- AGTGGCGCTGTAGTAGTTCTC-3’  5’-AAGGGGAATCGGATGGGGTT -3’  5’- TGCTCCGTATAGACCGGGAC-3’  5’- ACTGCCGCTATTCTTGTCCC-3’  5’- TCCCCCGGGGGATGGTGGTGGAGGA -3’ | RT-PCR  RT-PCR  RT-PCR  RT-PCR  RT-PCR  RT-PCR  RT-PCR  RT-PCR  RT-PCR  RT-PCR  RT-PCR  RT-PCR  RT-PCR  RT-PCR  RT-PCR  ChIP |
