# Supplementary material for: Notch3 Knockout Suppresses Mouse Mammary Gland Development and Inhibits the Proliferation of 4T1 Murine Mammary Carcinoma Cells via CCL2/CCR4 Axis
Source: Front Cell Dev Biol. 2020 Nov 17;8:594372. doi: 10.3389/fcell.2020.594372 (PMC7685216; doi:10.3389/fcell.2020.594372)
Supplement: Supplementary file 2 [file Table_2.docx]

**Supplementary Table 2. Antibodies used in this study**

| Antibody | Cat:# | Company | Con. | Species |  |
| --- | --- | --- | --- | --- | --- |
| CCL2 | BM1255 | BOSTER | 1:100 | Mouse | WB |
| CCL2 | BM1255 | BOSTER | 1:50 | Mouse | IHC-P |
| Notch3 | 55114-1-AP | Proteintech | 1:1000 | Rabbit | WB |
| Notch3 | #2889 | CST | 1:50 | Rabbit | CHIP |
| Rabbit (DA1E) mAb IgG | #3900 | CST | 1ug/1mg protein | Rabbit | CHIP |
| Tubulin alpha | AF7010 | Affinity | 1:5000 | Rabbit | WB |
| GAPDH | TA-08 | ZSGQ-BIO | 1:1000 | Mouse | WB |
