# Supplementary material for: Notch3 Knockout Suppresses Mouse Mammary Gland Development and Inhibits the Proliferation of 4T1 Murine Mammary Carcinoma Cells via CCL2/CCR4 Axis
Source: Front Cell Dev Biol. 2020 Nov 17;8:594372. doi: 10.3389/fcell.2020.594372 (PMC7685216; doi:10.3389/fcell.2020.594372)
Supplement: Supplementary file 3 [file Table_3.docx]

|  | **Notch3wt/wt (n)** | | | **Notch3wt/-** | | | **Notch3-/-** | | |
| --- | --- | --- | --- | --- | --- | --- | --- | --- | --- |
| **Aged** | **3w** | **5w** | **8w** | **3w** | **5w** | **8w** | **3w** | **5w** | **8w** |
| **Whole mount** | **3** | **3** | **3** | **3** | **3** | **3** | **3** | **3** | **3** |
| **RT-PCR** | **3** | **3** | **3** | **3** | **3** | **3** | **3** | **3** | **3** |
| **WB** |  |  | **3** |  |  | **4** |  |  | **5** |
| **Immunohistochemistry** | **3** | **3** | **3** | **3** | **3** | **3** | **3** | **3** | **3** |
| ***Intraductal Injection*** |  |  | **3** |  |  |  |  |  | **3** |
| ***Transcriptome profiling by RNA-Seq*** | **3** | **3** | **3** | **3** | **3** | **3** | **3** | **3** | **3** |
| ***Died pulps (newborn)*** | **0** | | | **6 (no milk in the stomach of the newborn pulps)** | | | **17(no milk in the stomach of the newborn pulps)** | | |

Supplementary Table3:

The numbers of the animals used and died in this study
