# Supplementary material for: Notch3 Knockout Suppresses Mouse Mammary Gland Development and Inhibits the Proliferation of 4T1 Murine Mammary Carcinoma Cells via CCL2/CCR4 Axis
Source: Front Cell Dev Biol. 2020 Nov 17;8:594372. doi: 10.3389/fcell.2020.594372 (PMC7685216; doi:10.3389/fcell.2020.594372)

Notch3 influences mouse mammary glands development and proliferation of 4T1 mammary tumor cells via CCL2/CCR4 axis

Supporting information:

Figure S1: The expression levels of CCL2 and its receptor CCR4 are different in various molecular subtype breast cancer, which are closely relate to the OS and DMFS of breast cancer patients.

(A): Boxplots on the upper row showing relative expression of CCL2 in basal, HER2 positive, luminal A, luminal B, normal-like, and unclassified breast cancer patients. Boxplots in the lower-left corner showing relative expression of CCL2 in ERα positive and negative status. Boxplots in the lower-right corner showing relative expression of CCL2 in breast cancer patients with grade 1, 2, and 3.

(B): cBioPortal's survival analysis shows that CCL2 is a significant and independent prognostic marker of overall survival in patients with breast cancer, and its high expression is correlated with poor overall survival (p=0.0202). (C): GOBO survival analysis shows that high CCL2 expression is correlated with poor DMFS (CCL2_low (log2 expression -5.822 to -0.463), CCL2_medium (log2 expression -0.463 to 0.656), and CCL2_high (log2 expression 0.656 to 6.386)) (p=0.00361).

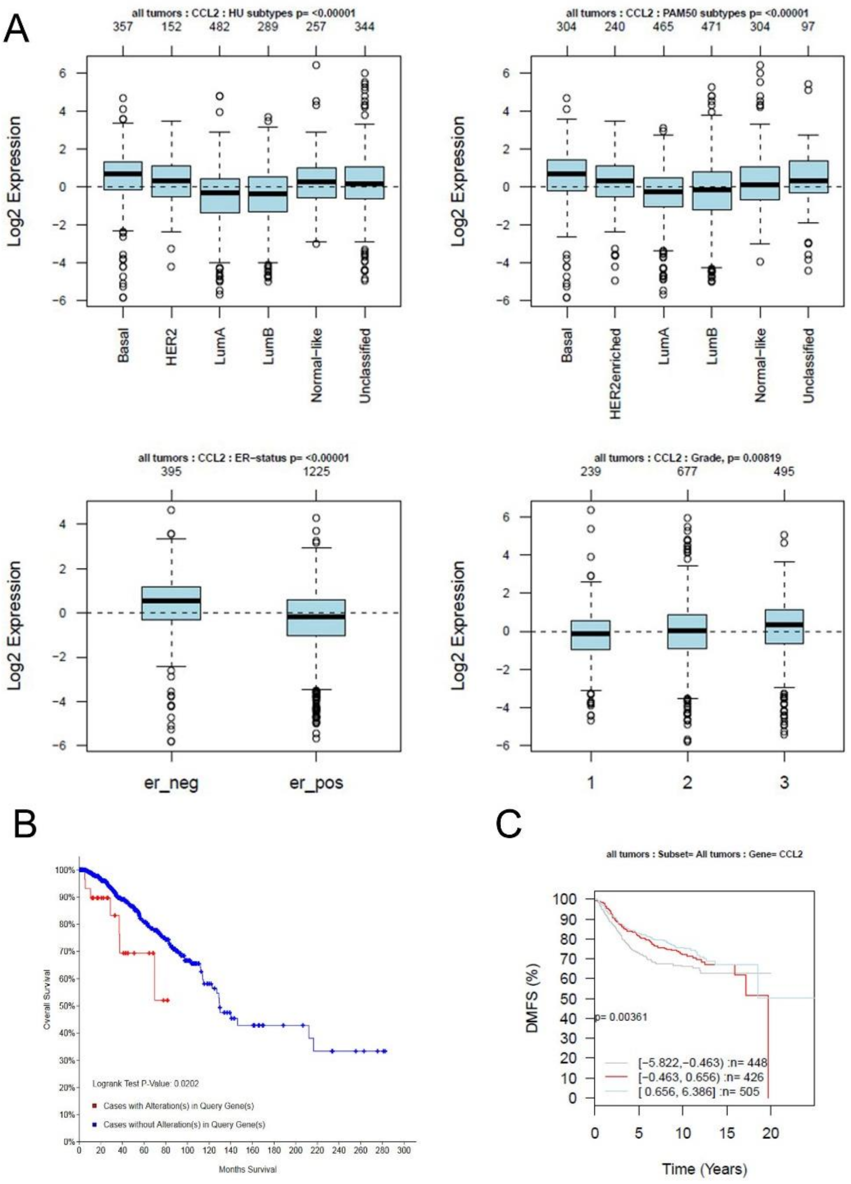

Figure S2

To ensure the firefly luciferase Luc2 gene to express, in vitro cell culture luciferase assay was carried out. The 293T cells were infected with control and CCL2 overexpression lentivirus respectively. Bioluminescence images were taken at 72h after infection. The results showed that the bioluminescent signals from 293T-luc2 and 293T-luc2-CCL2 cells were easily detectable, while the signal intensity signal decreased in turn due to a serial dilution concentration of the lentivirus. These data illustrate that Luc2 can express very well.

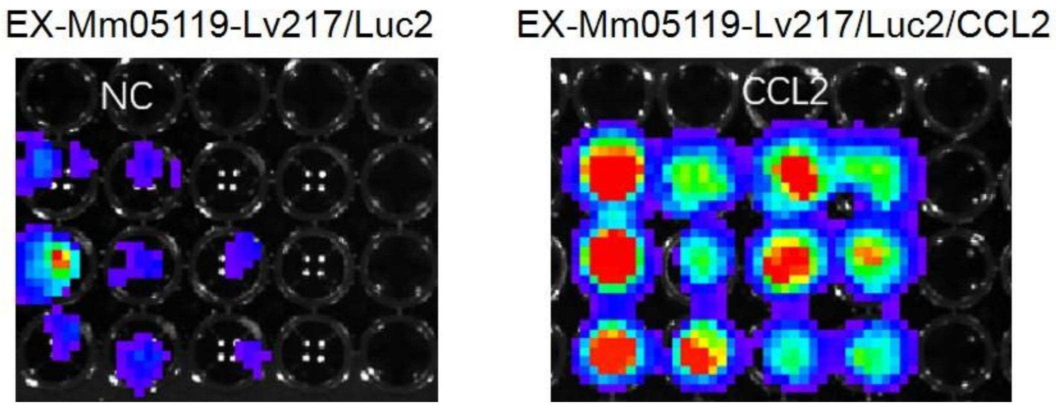

In vitro cell culture luciferase assay. 293T cells infected with EX-Mm05119-Lv217/Luc2 or EX-Mm05119-Lv217/Luc2/CCL2 respectively. In detail, 100 μl 293T cell suspension (12000 cells) was added into the wells of the sterile 96-well cell culture plate. Incubate the cells for 12 hours at 37°C (5% CO_2). Then, the cells were infected with a serial diluted concentration of the lentivirus, such as 1:1000, 1:2000, 1:4000, 1:8000. After 72h of infection, the luciferase activities were detected.

Figure S3

Practice injection: mammary gland injected with dye via intraductal injection. To test the accuracy of delivery via the nipple, a mouse at day 18.5 of pregnant was injected with 40 μl of trypan blue dye diluted in PBS. Following sacrifice, the gland was exposed for visualization. (A): bolus of dye at the nipple, and (B): successful injection (A)

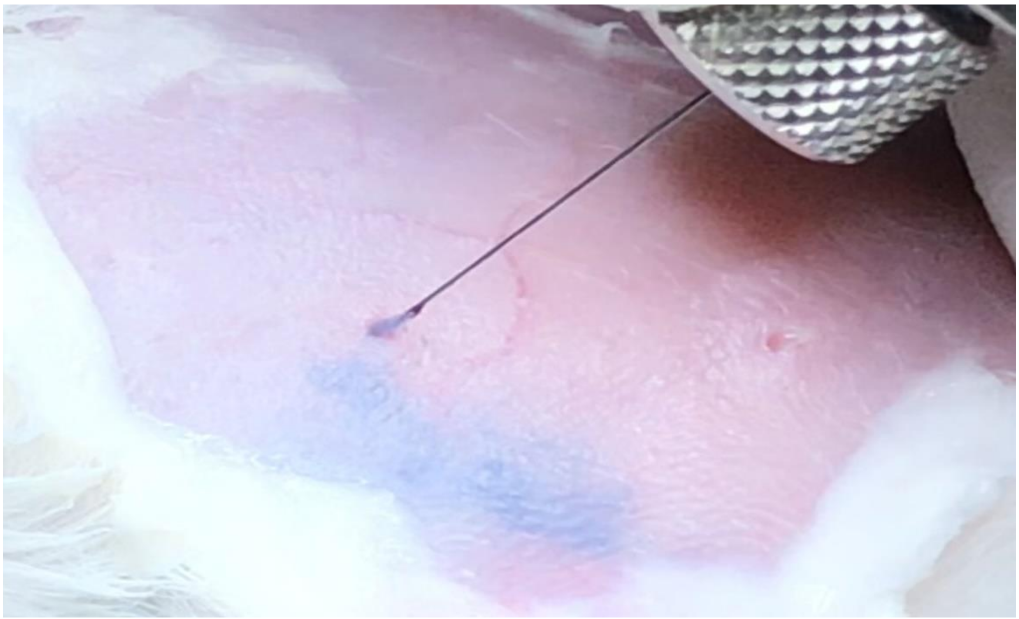

(B):

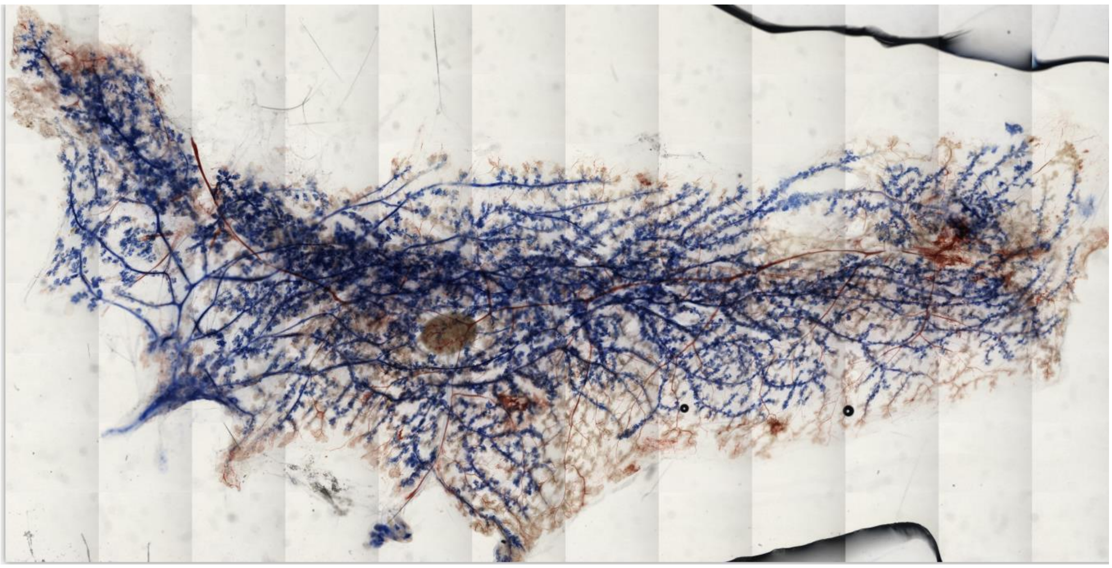

Figure S4:

It's hard to see the nipples from postnatal 8 weeks mice even under microscope.

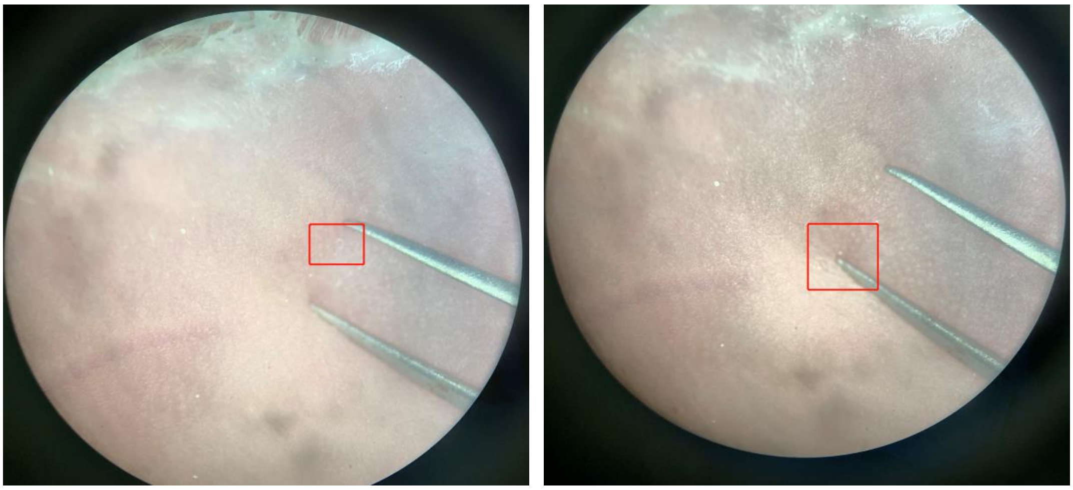

Supplement: Supplementary file 4 [file Data_Sheet_1.pdf]
