# Supplementary material for: Notch3 Knockout Suppresses Mouse Mammary Gland Development and Inhibits the Proliferation of 4T1 Murine Mammary Carcinoma Cells via CCL2/CCR4 Axis
Source: Front Cell Dev Biol. 2020 Nov 17;8:594372. doi: 10.3389/fcell.2020.594372 (PMC7685216; doi:10.3389/fcell.2020.594372)
Supplement: Supplementary file 5 [file Data_Sheet_2.pdf]

Supplementary Materials and Methods:

Quantitative real-time PCR

Total RNA was isolated from breast cancer cell lines using Trizol Reagent. First-strand cDNA was synthesized by incubating 1 µl of total RNA with oligo dT and reverse transcriptase (Superscript III, Invitrogen, Carlsbad, CA, USA), according to the manufacturer's protocol. Primers are listed in Supplementary Table 3. qPCR was performed using the Power SYBR Green PCR Master Mix (Life Technologies, Carlsbad, CA, USA) on the Bio-Rad 5-Color System (Bio-Rad, Hercules, CA, USA). *Gapdh* expression was used as an internal control for normalization. Relative changes in expression levels were calculated using the $2^{-(\Delta CT,Tg-\Delta CT,control)}$ method. All qRT-PCR analyses were performed in biological triplicates for each sample and a Student's *t*-test was used to determine statistical significance.

Western blotting

Protein lysates were extracted from breast cancer cell lines for immunoblotting as previously described. Briefly, protein lysates were separated and incubated with primary antibodies (Supplementary Table 1) overnight at 4°C, followed by incubation with horseradish peroxidase (HRP)-conjugated secondary antibodies (1:1000 dilution), with subsequent detection using the Pierce™ ECL substrate. Finally, PVDF membranes were scanned using the Quantity One Imaging system (Bio-Rad, Hercules, CA, USA) and densitometry ratios were obtained to semi-quantify relative expression levels of target proteins, and β-actin or GAPDH. Each experiment was repeated three times. All values are presented as means±SD.
